# Barriers and facilitators to the utilization of the intensive adherence counselling framework by healthcare providers in Uganda: a qualitative study

**DOI:** 10.1186/s12913-022-08495-0

**Published:** 2022-08-31

**Authors:** Pius Musinguzi, Josephine Nambi Najjuma, Adellah Arishaba, Eric Ochen, Racheal Ainembabazi, Fred Keizirege, Racheal Lillian Sabano, Edith K. Wakida, Celestino Obua

**Affiliations:** 1 Department of Nursing, Mbarara University of Science and Technology, Mbarara, Uganda; 2 Department of Medicine, Mbarara University of Science and Technology, Mbarara, Uganda; 3 Department of Pharmacy, Mbarara University of Science and Technology, Mbarara, Uganda; 4 Department of Medical Laboratory Science, Mbarara University of Science and Technology, Mbarara, Uganda; 5 Office of Research Administration, Mbarara University of Science and Technology, Mbarara, Uganda; 6 Department of Medical Education, California University of Science and Medicine, San Bernardino, CA USA; 7 Office of the Vice Chancellor, Mbarara University of Science and Technology, Mbarara, Uganda

**Keywords:** Intensive adherence counselling, Barriers and facilitators, Uptake and utilization, Health care providers, SURE framework

## Abstract

**Background:**

Uganda Ministry of Health (UMOH) embraced the World Health Organization recommendation for people living with human immunodeficiency virus with a detectable viral load (VL) exceeding 1000 copies/mL to receive intensive adherence counselling (IAC). The IAC framework was developed as a step-by-step guide for healthcare providers to systematically support persons with non-suppressed VL to develop a comprehensive plan for adhering to treatment. The objective of this study was to explore the current practice of the healthcare providers when providing IAC, and identify the barriers and facilitators to the utilization of the UMOH IAC framework at two health centers IV level in rural Uganda.

**Methods:**

This was a descriptive cross-sectional qualitative study that explored the current practices of the healthcare providers when providing IAC, and identified the barriers and facilitators to the utilization of the UMOH IAC framework. We used an interview guide with unstructured questions about what the participants did to support the clients with non-suppressed VL, and semi-structured questions following a checklist of categories of barriers and facilitators that affect ‘providers of care’ as provided by the Supporting the Use of Research Evidence for policy in African health systems (SURE) framework. Current practice as well as the categories of barriers and facilitators formed the a priori themes which guided data collection and analysis. In this study we only included healthcare providers (i.e., medical doctors, clinical officer, nurses, and counsellors) as ‘providers of care’ excluding family members because we were interested in the health system.

**Results:**

A total of 19 healthcare providers took part in the interviews. The healthcare providers reported lack of sufficient knowledge on the UMOH IAC framework; most of them did not receive prior training or sensitization when it was first introduced. They indicated that they lacked counselling and communication skills to effectively utilize the IAC framework, and they were not motivated to utilize it because of the high workload at the clinics compounded by the limited workforce.

**Conclusions:**

Although the UMOH IAC framework is a good step-by-step guide for the healthcare providers, there is need to understand their context and assess readiness to embrace the new behavior before expecting spontaneous uptake and utilization.

**Supplementary Information:**

The online version contains supplementary material available at 10.1186/s12913-022-08495-0.

## Background

Uganda Ministry of Health (UMOH) embraced the World Health Organization (WHO) recommendation for all people living with human immunodeficiency virus (PLHIV) with a viral load (VL) exceeding 1000 copies/mL to receive intensive adherence counselling (IAC) [1–4]. According to the WHO, VL failure is where there is a persistent detectable VL (two times consecutively when measured within a 3-month interval with adherence support) after at least 6 months of starting antiretroviral therapy (ART) [5–7]. In 2016, the consolidated guidelines for prevention and treatment of HIV in Uganda were developed with a framework on IAC to be followed [8]. Studies have shown that IAC achieves VL suppression in over 70.5% of PLHIV on ART with a non-suppressed VL [9–12]. Accordingly, based on the UMOH, VL monitoring was considered the gold standard for adherence and approving treatment response; therefore all PLHIV undergo a VL test 6 months after starting treatment, at 12 months and thereafter annually if the patient is stable on ART [5, 8].

The UMOH IAC framework was developed as a step-by-step guide for providers of care to systematically support people on ART with non-suppressed VL, and develop an inclusive treatment adherence plan [8]. It involves identifying and gaining insight into barriers to adherence, exploring possible ways to overcoming the barriers, and planning adherence to medical care [8, 13]. Pragmatically, ‘providers of care’ in IAC includes a multidisciplinary team of clinicians, nurses, counselors, family members, and peers [8].

### Intensive Adherence Counselling (IAC) framework

According to the consolidated guidelines for prevention and treatment of HIV in Uganda [8], the IAC framework consists of 5A’s which are methodically given for the healthcare providers to follow in a regular pattern (typically one month apart for three sessions) when dealing with a non-suppressed VL. The 5A’s include: *(1) Assess* –explaining purpose of session, disclosing the VL test results and what the implications are; determining the level of adherence and assessing possible barriers; *(2) Advise*—identifying gaps in client information, telling them the benefits of good adherence, and discussing possible consequences of non-adherence; *(3) Assist*—evaluating with the client the possible causes of the barriers and brainstorming possible strategies to overcoming them, and discussing the benefits of each option; *(4) Agree on* – the team agreeing on the way forward to address each key barriers, evaluating each action point while documenting the plan on the IAC session form which is filed for review in the next session; (5) *Arrange* – the healthcare provider finally recaps the session to the client, makes arrangement for ART refill, clarifies the IAC schedule to the client, and writes date on the next session form; requesting the client to bring it along with the remaining pills.

Although provision of IAC entails a multidisciplinary team (clinicians, nurses, counselors, family members, and peers [1, 5, 8], with a clear framework for IAC and support for clients with detectable VL, there is paucity of information about their utilization of the IAC framework and the factors affecting implementation [14].

Using the categories of barriers that affect implementation of policy options by providers of care, guided by the SURE (Supporting the Use of Research Evidence for policy in African health systems) framework [15], the purpose of this study was to explore the current practice of the healthcare providers when providing IAC, and identify the barriers and facilitators to the utilization of the UMOH IAC framework, two health center IV level in rural Uganda.

## Methods

### Study design and setting

This was a descriptive cross-sectional qualitative study that assessed the barriers and facilitators to the utilization of the UMOH IAC framework by the health care providers while following an interview guide with both unstructured (objective 1) and semi-structured questions (objective 2). We used a conversational approach with the participants during the interview, encouraging them to provide accurate information [16].

The study was conducted at two public HCs IV in Rukiga District, Southwestern Uganda in February 2022. Rukiga district is approximately 43 km (27 miles) by road, southwest of Ntungamo, along the Mbarara-Ntungamo-Kabale-Katuna Road – making it approximately 350 km from Kampala (capital of Uganda). At the time of the study, Rukiga district had 30 publicly owned health centers (HC) (i.e., 2 HCs IV, 7 HCs III and 21 HCs II), and no district hospital. The health cadres at HC IV level include medical officers (general practitioners), clinical officers (diploma level medical assistants), nurses, a counsellor, midwives, laboratory technicians and nursing assistants.

### Study participants and recruitment

Our participants were healthcare providers (medical doctors, clinical officers, nurses, and counsellors) at two public HCs IV in Rukiga district. We purposively planned to include all healthcare providers at the two health facilities who directly provided treatment, management and care for the people living with HIV (PLHIV); were willing to participate in the interviews, and provided a written informed consent. Additionally, the two HCs IV were also purposively selected because they were the highest level of public health care with services freely accessible to PLHIV in the area at the time. Laboratory staff and nursing assistants were excluded from the study because their functions are more supportive than direct care.

### Study procedure

In-depth interviews were conducted by a trained research assistant with a nursing science background, and the field notes taken by the lead author (PM) who at the time was under training and mentorship. Data were collected in February 2022, with each interview lasting between 30 – 40 min.

All participants were assured about confidentiality of their responses [17]. Informed consent was sought and the participant were told about their right to freely decide to participate, and to withdraw at any time without penalty.

### Data collection tool

An interview guide with both unstructured and semi-structured questions was developed by PM in consultation with EKW (implementation scientist) and JNN (qualitative researcher). On current practices, we asked unstructured questions about the participants understanding of IAC and what they typically did to support the clients who had a non-suppressed VL; while for the barriers and facilitators, semi-structured questions were developed following three categories (knowledge and skills; attitudes regarding program acceptability, appropriateness, and credibility; and motivation to change or adopt) in the SURE framework- Supporting the Use of Research Evidence for policy in African health systems checklist for identifying barriers and facilitators for implementing policy options at the ‘Providers of care’ level [15] (Appendix file 1). The last author (CO)—a senior researcher on the team reviewed the tool for clarity, ensuring that it is defendable.

### Data collection

Data were collected from 19 out of the 22 eligible participants at the two health facilities in Rukiga district (9 were from one HC IV and 10 from the other HC IV). Three healthcare providers did not take part in the study because they were either on leave, away on official assignments or declined to take part citing personal reasons. Interviews were conducted in private spaces (offices) at the health facilities after the healthcare providers had finished attending to their clients. All interviews were conducted in English the official national language and audio recorded to crosscheck the information collected.

### Data management and analysis

The first two interviews were transcribed verbatim by the research assistant, and the transcripts checked by EKW for correctness of information before proceeding to the next set of interviews. This was done to ensure that the questions were being asked and responded to in the correct way and would answer the research questions. We used deductive thematic analysis for both the current practice and the barriers and facilitators. Current practice as well as the categories of barriers and facilitators that affect ‘providers of care’ as provided by the SURE framework checklist formed the a priori themes. Data were manually grouped and analyzed by PM, AA, FK, RA, EO and RLS under three categories i.e., knowledge and skills; attitudes regarding program acceptability, appropriateness, and credibility; and motivation to change or adopt new behavior. EKW and JNN independently read through the transcripts and developed codes under each category, did the first coding, and then discussed with CO before involving the entire study team (PM, AA, FK, RA, EO, RLS and CO) who would go on to complete the coding process. The coding was done iteratively to agree on the fit of response categorization.

## Results

Of the 19 participants interviewed (9 were from one HC IV and 10 from another), three were medical doctors, one clinical officer, four nurses at different levels of practice, nine midwives and two counsellors (Table 1). The participants had varying educational backgrounds ranging from bachelor’s degree as the highest level of education attained to certificate level. Five participants had less than 5 years of experience in healthcare service, nine between 5–10 years, and five had more than 10 years.

**Table 1:** Participants demographics

Participants	Age	Sex	Health cadre	Educational Level	Service Years
01	27	F	Counsellor	Diploma	5–10
02	29	F	Senior Nursing Officer	Degree	5–10
03	35	F	Enrolled Midwife	Certificate	5–10
04	25	F	Enrolled Midwife	Certificate	< 5
05	40	M	Senior Nursing Officer	Degree	> 10
06	33	F	Registered Midwife	Diploma	5–10
07	39	F	Registered Midwife	Diploma	> 10
08	26	F	Enrolled Midwife	Certificate	5–10
09	26	M	Medical Doctor	Degree	< 5
10	40	F	Registered Midwife	Diploma	> 10
11	40	M	Counselor	Degree	5–10
12	39	F	Registered Midwife	Diploma	> 10
13	35	F	Nursing Officer	Diploma	5–10
14	29	F	Enrolled Midwife	Certificate	5–10
15	40	M	Nursing Officer	Diploma	> 10
16	44	M	Clinical officer	Diploma	< 5
17	26	F	Enrolled Midwife	Certificate	< 5
18	34	M	Medical Doctor	Degree	5–10
19	35	M	Medical Doctor	Degree	< 5

### Current practice of the health care providers *(Objective 1)*

Prior to identifying the barriers and facilitators to the utilization of the UMOH IAC framework, we asked the participants questions about their understanding on IAC and what they typically did to support the clients who had a non-suppressed VL. Most participants reported knowledge about IAC, what was supposed to be done and what they were doing as exemplified below:

*Intensive adherence counselling, is the counselling that we are offering to clients with non-suppressed viral load. It is done for 3 months and then we repeat the viral load test. If it is not suppressed, we continue counselling the client for another 3 months as we are monitoring (Participant 8, Female Midwife, health facility 1)*.

In their narratives, most of the participants noted that IAC was meant for only the HIV clients with non-suppressed VL, to closely monitor challenges adherence to medications and provide psychosocial support.

*When we get clients for HIV, we first look at how they are taking their medications and how they are adhering to them. When we do the viral load testing and find that they are not doing well [unsuppressed], we sit with them and do counselling for three consecutive months…then we give them a return date, when we see that they have improved, we continue like that. But you know that this is done for those ones who are not suppressing, when the viral load keeps high we intensify with the counselling (Participant 5, Male Nursing Officer, health facility 2)*

However, one participant had a different school of thought that called for giving IAC to all HIV positive clients regardless of the VL status. The argument put across was the need to motivate the clients with suppressed VL to keep doing so.

*For me, I do intensive adherence counselling for every interface with the client whether non-suppressed or suppressed… because even the suppressed, we need to encourage them to keep doing good adherence to drugs. So, for me when dealing with every client I consult the IAC guidelines (Participant 7, Female Midwife, health facility 1).*

The participants alluded to the fact that the clients’ responses were dependent on how IAC was presented to them, and that it would either motivate them to pick or lose interest as illustrated below:

*They receive it well, actually they like it depending on how you have explained it…I tell them how the viral load is too high and what it means, for example that they will likely fall sick all the time and get diseases like meningitis... by doing so, I am provoking the client to begin asking the solution, they ask if it is too late to do anything…that way, I have created interest (Participant 2, Male Counsellor, health facility 1)*

Some participants highlighted that IAC provided them with opportunity to interact with the HIV clients to the extent that would otherwise not be possible during routine care. That during the engagement, the clients were able to open up on struggles that they had in the quest to find a solution.

*When you talk with those clients, they become free and tell you their problems. If they are taking drugs they will tell you how they are taking them, and sometimes they tell you their problems if they are not taking drugs so that you help them to overcome and they suppress (Participant 3, Female Midwife, health facility 2)*

### Barriers and Facilitators *(Objective 2)*

#### Category 1: knowledge and skills

In this category, most of the participants were uncertain about the framework that they were using to provide IAC, however, some of them indicated that IAC could be guidelines from the Ministry of Health:

*We are using Ministry of Health guidelines, most likely the consolidated HIV guidelines 2020…Ahha, mostly what is entailed there is one, initiation of ARV’S or ART to these clients; two, the package we should give to these HIV clients; and then counselling which includes this intensified counselling (Participant 5, Male Nursing Officer, health facility 2).*

Most of the participants made a comparison between the UMOH IAC framework and what they were initially following from the implementing partners. From the participants view point at the time of the study, the UMOH framework did not provide them the flexibility to record the barriers identified and then plan on addressing them to enable follow-up at the next visit.

*We used to have printouts of IAC forms provided by HIV implementing partners on how to do it and when to end the session. However, now the Ministry of Health changed by putting it in the blue card, but it is not helping us so well like the other one… the original forms had where to write the barriers, and how they were addressed; the blue card does not provide for that. Initially, any clinician would follow-up on the barrier recorded on the form, but with the blue card there is no continuity (Participant 2, Male Counsellor, health facility 1).*

Most of the participants reported not receiving specific training on implementation of the UMOH IAC framework. They highlighted that when there was opportunity for training, only one representative was selected per health facility to be trained as a trainer so that they would conduct facility-based training. However, at one health facility, it was reported that the trained person was transferred to another health site before training at the facility could take place.

*I have not received any training on the IAC framework…there was one training where one person from this facility attended, after the training he was transferred to another facility [Laughs]…so, we’ve not had these trainings (Participant 10, Male Doctor, health facility 1).*

The participant who reported receiving training noted that it was generalized to HIV and that it was provided by implementing partners. She added that there was a component of intensive adherence counselling and listed the sequence to be followed, but with no specific details of what was to be done.

*The implementing partners trained us on some guidelines the other year but we don’t still remember. On the flip chart they used, there was that part for counselling… They told us that when someone is non-suppressing you give three sessions; you counsel first month, second month and third month then you take the sample for repeat. That’s what I picked (Participant 3, Female Midwife, health facility 2)*

All the participants indicated that they needed skills in counselling to implement the UMOH IAC framework. Some of them reported that in scenarios where a client needed intensive adherence counselling, they called in a counsellor to support the session because they did not feel competent to handle the process.

*…we all need to have counseling skills. It's something not easy…actually most times when I am in ART clinic and I identify a patient who requires intensive adherence counselling, I engage the counsellor. I feel I cannot like do it alone, you know…. So, I most of the time involve the counsellor (Participant 9, Male Doctor, health facility 1).*

Some participants added that communication skills were also needed; that having knowledge without knowing how to communicate the message to the clients was not helpful. They highlighted the need for the health care providers to learn how to communicate sensitive information to the clients to avoid unintended responses.

*The skill of communicating is very important, sometimes you have the knowledge but cannot pass it on. Communication is very vital when talking to a client, for example, sometimes you can ask a question that may provoke the client from answering you, or she even gives you a wrong answer that you had not expected because of the question you asked her. Maybe the question sounded rude… (Participant 6, Female Midwife, health facility 2).*

#### Category 2: attitudes regarding program acceptability, appropriateness, and credibility

In this category, most of the participants who reported using the UMOH IAC framework found it useful when providing counselling and highlighted the need to train other health care providers to use it. They said that using the framework well, when providing the counselling, had the potential to reduce non-adherence to ART.

*Actually, this protocol [UMOH IAC framework] is the best I can say if well implemented...I am very sure that if it is emphasized and used very well, we shall no longer have poor adherence cases in ART. (Participant 1, Female Midwife, health facility 2).*

*I think the protocol [UMOH IAC framework] is good and most people benefit from it, but it needs training of many health workers to have the knowledge because we have a knowledge gap on how to use it (Participant 10, Male Doctor, health facility 1).*

The participants thought that the UMOH IAC framework was appropriate and acceptable and that it helped them to get information from the clients which they would otherwise not have been able to get. One participant specifically noted that all health care providers in their setting interfaced with a client who was living with HIV, that it was important for each one of them to interest themselves with what the UMOH IAC framework said and implement it since it was not realistic to leave all the clients needing IAC to the few counsellors.

*Right now, I cannot say it is not appropriate because it is helping our clients to tell us issues which we’ve not been knowing about. The protocol [UMOH IAC framework]is relevant, because I don't think that there is any health worker who can spend two days without seeing a HIV positive client. We have very many [HIV positive clients] and we cannot say we leave them to one person may be the counsellor. All of us have to be responsible and have to implement it…we should not say that it is inconveniencing because it is almost part of routine work (Participant 6, Female Midwife, health facility 2).*

All participants reported development of sustained client relationships, beyond the struggles of viral load non-suppression, to continued engagement. They added that this relationship reduced instances of future failed viral load non-suppression.

*This direct contact with the client brings about a good relationship…when I discuss with a client two to three times, even after suppression, there is continuity of that relationship. You find the client calling you…You know, HIV is still there, even stigma, but we are trying to reduce. Even after the sessions end and the patient’s viral load is suppressed, the rapport is maintained, and it prevents subsequent viral load non-suppression of the client (Participant 7, Male Clinical Officer, health facility 1).*

In relation to what did not work well, the participants who were implementing the UMOH IAC framework noted that they largely offered facility-based counselling because of the challenges involved with home visits including lack of transportation. As a result, they missed to identify family-based challenges which largely contributed to the viral load suppression status.

*What has not work well for us is that, we are counselling some of the people at the facility level and we are not following them at the community level because we do not have [transport] facilitation. Most of the problems are at community level…issues at family level. So, I think we need to strengthen that community-based visits, so that we finish those issues (Participant 6, Male Nursing Officer, health facility 1).*

Most participants indicated that most of the clients arrived to the health facilities late because of the distances from their homes, and that they arrived when hungry so their concentration span was very short. It would become difficult to use the framework in such a situation.

*…sometimes you find that the client doesn’t come in time they come late, and yet we have other patients to see… finding time to talk to other patients becomes difficult…Secondly some clients come from very far and when they reach, you find that they are tired and hungry… people are very poor here so you find even if you’re talking to someone, they are not minding about what you are saying (Participant 5, Male Nursing Officer, health facility 2).*

#### Category 3: motivation to change or adopt new behavior

In this category, most participants indicated that seeing clients improve, and the clients recognizing their efforts was a motivation for them to use the UMOH IAC framework. One participant added that with this effort came the ability to identify clients who were developing resistance to ART.

*… am happy when these people are taking their medication very well. This person is coming and appreciating, and people are healthy… that one has motivated me to like this protocol [the UMOH IAC framework]. Fewer people are being changed from first line to second line…it has helped us to know people who are getting resistant to these drugs (Participant 5, Male Nursing Officer, health facility 2).*

Some participants reported that the UMOH IAC framework was a one-stop shop for all the procedures when supporting clients with viral load non-suppression, and they were motivated to use it as illustrated in the quote below.

*That tool [UMOH IAC framework] guides me on the key things, and without it I may forget somethings because I don’t write them. The tool guides me on how to identify those factors that cause [viral load] non-suppression...basically, I use it because I want to see our clients suppressed (Participant 10, Male Doctor, health facility 1).*

All the participant midwives in the study were motivated to use the UMOH IAC framework because they understood its importance in protecting the infants from being infected with HIV by their mothers at birth.

*We midwives are aware of this tool and the importance, we strongly accept the use of it. For the mothers, we know the importance of it protecting their unborn infants so we educate them. It [the UMOH IAC framework] is good, because it helps on improving the quality of the services we are offering and the health status of the client. We receive mothers during antenatal, who are non-suppressed [viral load] and some who are newly diagnosed (Participant 8, female enrolled midwife, health facility 1).*

Some participants reported that using the UMOH IAC framework was time consuming and was not practical for medical doctors because they provided oversight to all health care delivery at the health facilities including medical emergencies. One participant was particularly not motivated about starting to engage with a client on IAC and midway drops out to attend to the other medical emergencies, he preferred not to start at all as illustrated in the quote below.

*A single client requires a lot of time for IAC…assuming you had more than one, you only do that for the whole day. Now for a medical officer, assuming you are giving IAC and they call you to other departments in the health facility to attend to an emergency, what happens to the client? I wouldn’t want to do that (Participant 10, Male Doctor, health facility 1).*

Participants highlighted the need for more counsellors and training of health care providers to use the UMOH IAC framework. They reported that with the high volume of clients and a limited health workforce to provide the service, it was strenuous to offer IAC as required. They recommended provision of funding for home-visits to reduce the patient workload at the health facilities.

*I think, if we are given funds, we identify those who are not suppressing, we follow them up, and we make action plan with them at home, I think that would help us. We need to have additional counselors because the clients are many…we have one counselor, that is not enough. And for us who would support him, we are also strained by other duties. So, you find most of the times he is alone…of course he tries his level best, but he needs support (Participant 6, Male Nursing Officer, health facility 1).*

## Discussion

In this study, we were interested in understanding the current practices of the healthcare providers in supporting clients with a non-suppressed VL; and the barriers and facilitators to utilization of the UMOH IAC framework, with the goal to find a solution to the barriers and promote behavior change or attitudes that would facilitate uptake [18].

Clinical guidelines are developed with the intention of standardizing procedures followed and improving patient outcomes [18]; they summarize evidence to inform clinicians’ decision making, but how they are developed and written influences how often they are used [19]. Procedurally, we used the SURE framework to guide the data collection and analysis with focus on three specific categories of barriers and facilitators that health care providers typically face when implementing policy options in African health systems [15]. Increasingly, researchers in sub-Saharan Africa are adopting the use of the SURE framework; the closest examples being a systematic review by Gugulethu et al. 2020 where the researchers were looking at barriers and facilitators of rendering HIV services by community health workers in sub-Saharan Africa [20], and Wakida et al. 2019 looking at Health system constraints in integrating mental health services into primary healthcare in rural Uganda [21].

Like elsewhere [22, 23], studies have been conducted in Uganda to assess implementation of IAC from the lens of the health care providers (family and health workers) [11, 24, 25], and PLHIV [26, 27]. In this study we focused on the healthcare providers and explored the barriers to the utilization of the UMOH IAC framework in order to identify potential solutions that may be more broadly applicable and feasible [19]. Of the three categories of barriers and facilitators, the ‘knowledge and skills’ category emerged strongest and spilled into the other categories of barriers.

### Knowledge and skills

All the health care providers at the two health facilities perceived the UMOH IAC framework as useful and were willing to use it during the IAC sessions. However, there was a knowledge and skills gap in the utilization of the UMOH IAC framework; this is a recipe for failure as highlighted in the results. It is difficult for the clients to follow instructions from the health care providers if they do not know how to communicate to the clients. This finding is similar to another study conducted in Uganda on the experience of IAC in a public health center in Kampala [11]. Additionally, sensitization prior to introducing an intervention is key in disseminating evidence-based practice [28]. According to our study, there was insufficient dissemination and or sensitization of the UMOH IAC framework to the intended users at the health facilities. Most of the participants at the time of our study had scanty information on the UMOH IAC framework, thus, begging the question of how much uptake and utilization should be expected. In a study conducted in South Africa by McCaul et al. 2019, it was reported that the lack of consistent and clear communication from regulators regarding the guidelines, career pathways, and up-skilling had left health care providers confused [28].

### Attitudes regarding program acceptability, appropriateness, and credibility of the UMOH IAC framework

The participants largely had a positive attitude as illustrated in the results, what stood out the most was the client’s openness to the health care providers about the barriers they faced leading to the VL non-suppression status, the eventual sustained patient-provider engagement, and the fact that there was potential to prevent future non-suppression with sustained communication. The finding on positive attitudes of the health care providers is comparable to that of McCaul et al. 2019 wich highlighe the benefit of establishing rapport with the clients [28]. This however means that more training on identified gaps, in counselling and communication, can be useful in strengthening acceptability, appropriateness and credibility and can subsequently reduce the burden on the people currently providing IAC.

### Motivation to change or adopt a new behavior

While the majority of the participants were motivated to implement the IAC or were willing to change or adopt new behavior, time commitment, work overload, limited workforce, and few counsellors were demotivating factors in this category of barriers, thus, challenging the health care providers readiness to adopt the behavior of utilizing the UMOH IAC framework. To understand behavior change related to uptake of guidelines, assessing the readiness to change of the health care providers needs to be considered, as it cannot be assumed that all health care providers are similarly motivated to embrace the practice [29]. In a study conducted by Stander et al. 2021, where a model of time management for better clinical practice guidelines uptake was tested, it was determined that when clinicians can identify their level of readiness to use the guidelines, then they are able to choose the strategy to enable moving forward and improve or maintain their guideline uptake [29]. From our study, the participants recommended two strategies that would motivate utilization of the UMOH IAC framework, which were, recruitment of more counsellors at the health facilities to provide IAC, and providing training to all the health care providers so that they could have the skills to provide IAC using the available framework. Although there is evidence that IAC is a cornerstone for improved adherence and better health outcomes [30], there is need to understand context specific cultures and circumstances before developing different strategies [31]. Adherence to ART is a life-long requirement, there is need to concentrate on improving existing services and provide them in a more holistic way to improve outcomes than introducing new strategies [32].

#### Strengths and limitations

The strengths of this study included the use of the SURE framework to guide the identification and analysis of the barriers that affect implementation of policy options in the African health system. Participants were from the two highest level of health care provision (HC IV) in a rural district setting and represented the full spectrum of health care providers’ experience and demographics. The questions were designed along the barrier categories as enlisted in the SURE framework to capture all relevant areas.

Qualitative studies are subjective in nature, however, we tried to be as objective as possible when asking our questions to pick the context specific factors from the perspective of the participants. Additionally, although not generalizable, the responses from our study may be transferable to other districts with similar contexts for comparison. The findings from this study will provide a basis for future work.

## Conclusions

Although the UMOH IAC framework was a step-by-step guide for the providers of care, there was need to understand the contextual and practical challenges of the healthcare providers to assess their readiness to embrace the new behavior of utilizing the framework. From our study, the health care providers (a) lacked sufficient knowledge on the UMOH IAC framework because most of them did not receive prior training or sensitization, (b) lacked counselling and communication skills to effectively utilize the IAC framework, and (c) were not motivated to utilize the UMOH IAC framework because of the high workload at the clinics compounded by the limited workforce. However, they had a positive attitude towards the outcome of utilizing the UMOH IAC i.e., understanding their clients from a personal point of view and establishing rapport that supports them through the adherence struggles, and keeping hopeful that once their knowledge and skills are enhanced, then they could provide IAC to the clients.

These findings are confirmation that there is no ‘one-size fits all’ when designing practice guidelines, thus, the need to consider the practice environment of the intended implementer and readiness to adopt a new intervention before expecting spontaneous uptake and utilization.

## Supplementary Information

**Additional file 1.**

## Data Availability

Data on which this manuscript is based will be available from the corresponding author on reasonable request.

## Abbreviations

ART: Antiretroviral therapy
GUREC: Gulu University Research Ethics Committee
HEPI-TUITAH: Health Professional Education Partnership Initiative –Transforming Ugandan Institutions Training Against HIV/AIDS
HIV: Human Immunodeficiency Virus (HIV)
IAC: Intensive Adherence Counselling
SURE: Supporting the Use of Research Evidence
UMOH: Uganda Ministry of Health
UNCST: Uganda National Council for Science and Technology
WHO: World Health Organization (WHO)
VL: Viral load
