# Supplementary material for: Barriers and facilitators to the utilization of the intensive adherence counselling framework by healthcare providers in Uganda: a qualitative study
Source: BMC Health Serv Res. 2022 Aug 31;22:1104. doi: 10.1186/s12913-022-08495-0 (PMC9429278; doi:10.1186/s12913-022-08495-0)
Supplement: Supplementary file 1 — Additional file 1. [file 12913_2022_8495_MOESM1_ESM.docx]

**IN-DEPTH INTERVIEW GUIDE**

**BARRIERS AND FACILITATORS TO THE UTILIZATION OF THE INTENSIVE ADHERENCE COUNSELLING FRAMEWORK BY PROVIDERS OF CARE IN RUKIGA DISTRICT, SOUTH-WESTERN UGANDA: A QUALITATIVE STUDY**

**Interview guide ID…………………………………Date of the interview………………………** Introduce yourself……………………………………………………………………………………. **Socio-demographic characteristics**

1. Name of facility………………………………………………
2. Age……………………………………………………………
3. Gender: Male………Female……….
4. Profession………………………………………………………….
5. Level of professional education……………………………………………
6. Years in practice……………………………………………...

**Current practice**

1. Please tell me about IAC and how you are doing it

2. How do you think it has been received by the clients?

**Knowledge and skills**

3. What guidelines are you using to provide IAC?

4. What is your experience with the MOH IAC protocol?

5. Tell me about the training have you received in the implementation of the MOH protocol on IAC

6. What skills do you think are needed to implement the MOH IAC protocol?

**Attitudes regarding programme acceptability, appropriateness and credibility**

7. What is your opinion about the MOH IAC protocol?

8. How acceptable/appropriate/relevant is the MOH IAC protocol in relation to your work environment?

9. What has worked well for you when using the MOH IAC protocol?

10. What has not worked well for you when implementing the IAC protocol?

11. How often do you refer to the MOH IAC protocol?

12. What challenges have you encountered with following the IAC protocol?

13. How best do you think the MOH IAC protocol should be used?

14. How best should providers of care be supported to utilize the MOH IAC protocol?

15. What do you think needs to change in the protocol for better outcomes?

**Motivation to change or adopt new behaviour**

16. Please tell me about your motivation to use the MOH IAC protocol

17. How does it affect your work as a health care provider in HIV service delivery?

18. In your opinion, how do your clients receive the IAC provided to them?

19. How does that motivate you to carry on with IAC?
